# Extensive myocardial calcification following cytokine release syndrome and sepsis: a novel case report with advanced echocardiographic assessment

**DOI:** 10.1093/ehjcr/ytag071

**Published:** 2026-02-18

**Authors:** Lucía Canales-Muñoz, Clara Ugueto-Rodrigo, Carlos Rodríguez-Carneiro, Alejandro Lara-García, Leonel Diaz-González, Raúl Moreno

**Affiliations:** Cardiology Department, La Paz University Hospital, IdiPAZ, P° de la Castellana 261, 28046 Madrid, Spain; Cardiology Department, La Paz University Hospital, IdiPAZ, P° de la Castellana 261, 28046 Madrid, Spain; Cardiology Department, La Paz University Hospital, IdiPAZ, P° de la Castellana 261, 28046 Madrid, Spain; Cardiology Department, La Paz University Hospital, IdiPAZ, P° de la Castellana 261, 28046 Madrid, Spain; Cardiology Department, La Paz University Hospital, IdiPAZ, P° de la Castellana 261, 28046 Madrid, Spain; Cardiology Department, La Paz University Hospital, IdiPAZ, P° de la Castellana 261, 28046 Madrid, Spain

**Keywords:** Case report, Myocardial calcification, Cytokine release syndrome, Global longitudinal strain, Myocardial work, Sepsis, Advanced echocardiography

## Abstract

**Background:**

Myocardial calcification is a rare complication, typically associated with chronic myocardial injury or severe metabolic disturbances. Rapid development in the setting of cytokine release syndrome (CRS) and sepsis is exceptional and, to our knowledge, has not been previously reported.

**Case summary:**

We describe a 44-year-old man with acute myeloid leukaemia who developed CRS and subsequent septic shock, requiring high-dose vasopressor support and continuous renal replacement therapy. On day 70 of hospitalization, a computed tomography scan revealed extensive intramyocardial calcification of the left ventricle. Serial transthoracic echocardiography demonstrated progressive systolic dysfunction, with global longitudinal strain deteriorating from −22% to −9%, and a markedly reduced global work index (GWI 780 mmHg%), indicating impaired intrinsic contractility. Global work efficiency (GWE 96%) remained preserved.

**Discussion:**

This is the first reported case of rapidly progressive myocardial calcification associated with CRS, documented using advanced non-invasive imaging modalities such as GLS and myocardial work. These techniques enabled early detection and objective assessment of functional decline in a critically ill patient, in whom conventional diagnostic options were limited.

Learning points

- Rapid myocardial calcification can occur following cytokine release syndrome, particularly when compounded by sepsis and multiorgan dysfunction.
- Global longitudinal strain (GLS) and myocardial work (MW) allow early detection and quantification of ventricular dysfunction in critically ill patients.
- Intense inflammation, catecholamine exposure, microvascular injury, and metabolic disturbances synergistically contribute to dystrophic myocardial calcification.

## Introduction

Myocardial calcification is an uncommon condition that can be classified as either dystrophic—the most frequent form, secondary to prior cellular injury—or metastatic, related to disturbances in calcium-phosphate metabolism.^1^ Currently, severe sepsis is recognized as one of the most common causes of dystrophic myocardial calcification.^2^

Cytokine release syndrome (CRS) is a potentially life-threatening systemic inflammatory reaction, usually triggered by immunotherapy, and characterized by fever, hypotension, multiorgan dysfunction, and marked elevation in interleukin-6 (IL-6).^3^ Although cardiac involvement in CRS has been described, its association with myocardial calcification had not been previously documented, and may be mediated by a massive inflammatory response leading to microvascular dysfunction and cellular necrosis.

In critically ill patients, advanced echocardiographic tools such as global longitudinal strain (GLS) and myocardial work (MW) enable the early detection of subclinical systolic dysfunction and provide a more accurate, load—adjusted assessment of myocardial contractility—even in the setting of high afterload from vasopressor support.

## Summary figure

**Figure:**
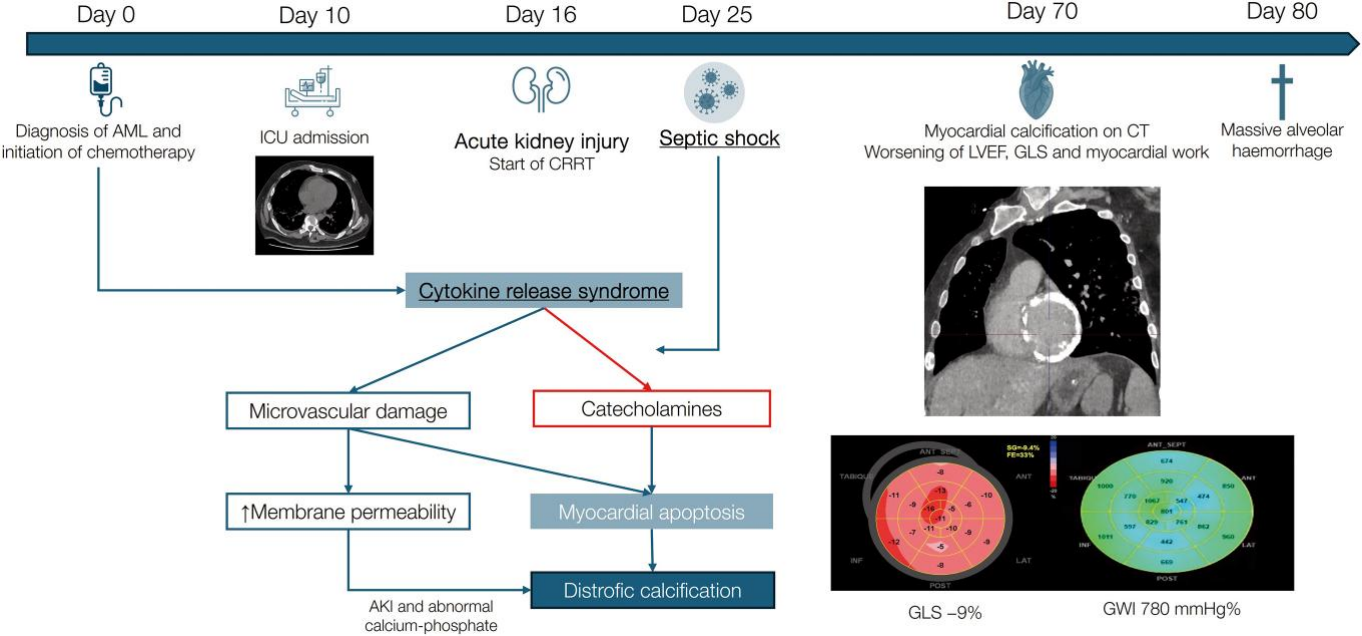
Timeline and pathophysiological mechanism of rapid myocardial calcification in citokine release syndrome (CRS). Timeline of key clinical events, from AML diagnosis (Day 0) and chemotherapy–induced CRS through ICU admission, acute renal failure with CRRT initiation, septic shock, to the diagnosis of extensive myocardial calcification on Day 70 and fatal alveolar haemorrhage on Day 80. Integrated pathophysiological schema shows how CRS and subsequent septic shock induce microvascular injury, increased membrane permeability, and myocyte apoptosis—driving dystrophic calcification—while acute renal failure with hyperphosphatemia contributes to calcific deposition. CT at Day 70 demonstrates marked left ventricular calcification; corresponding bull’s-eye plots reveal progressive decline in GLS (from −22% at baseline to −9% at Day 77) and GWI (780 mmHg% at Day 77), confirming intrinsic contractile deterioration.

## Case presentation

A 44-year-old male, active smoker, was admitted to our hospital following a diagnosis of intermediate-risk acute myeloid leukaemia (NPM1 and IDH2 mutations). Induction therapy with idarubicin, cytarabine, and gemtuzumab was initiated. From the onset of chemotherapy—particularly after gemtuzumab administration—the patient developed intermittent low-grade fever and systemic inflammatory symptoms, which required a reduction in the infusion rate of chemotherapy. Initially, these episodes were controlled with antipyretics. Laboratory tests revealed progressive pancytopenia and increasing levels of C-reactive protein (CRP), peaking around 30 mg/dL. A baseline transthoracic echocardiogram (TTE) showed no significant abnormalities, with preserved left ventricular ejection fraction (LVEF) and a GLS of −22% (see Supplementary material online, *Video S1*).

**Figure 1:**
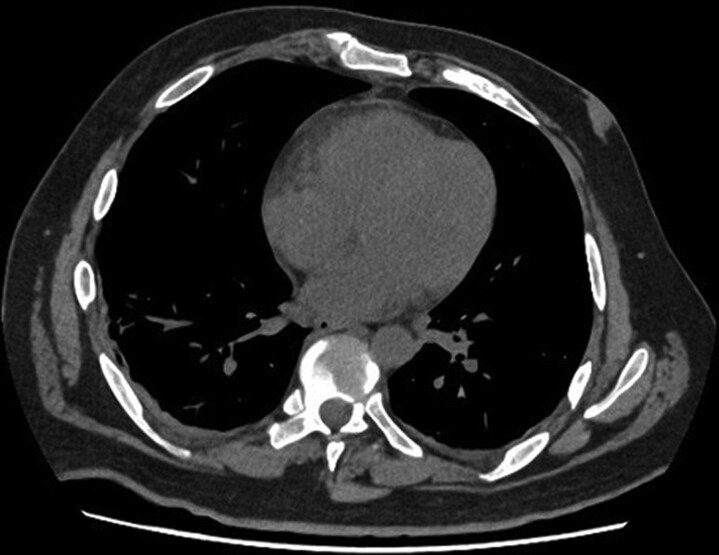
Baseline non-contrast chest CT on day 11 of hospitalization. Axial view showing no evidence of myocardial calcification. The left ventricular wall appears normal, without abnormal density or hyperattenuation.

On day 10 of induction, the patient was transferred to the intensive care unit (ICU) due to refractory hypotension requiring high-dose vasopressor (norepinephrine 0.5 µg/kg/min and vasopressin 1 U/h), in the setting of sustained fever (38.5°C) and respiratory deterioration requiring high-flow nasal cannula oxygen therapy. Laboratory findings showed profound neutropenia, a marked rise in CRP (up to 100 mg/dL), procalcitonin (PCT 40 ng/mL), hyperfibrinogenaemia (850 mg/dL), and significantly elevated IL-6 levels (1000 pg/mL; normal <15 pg/mL), along with renal and hepatic dysfunction. Chemotherapy was suspended due to haemodynamic instability and suspected CRS, supported by the rapid clinical deterioration, the inflammatory profile, and the absence of microbiological growth at that time. High-dose immunomodulatory therapy was initiated, consisting of three doses of intravenous methylprednisolone (500 mg/day) followed by a tapering course of dexamethasone, and two doses of tocilizumab. Empirical broad-spectrum antimicrobial therapy was maintained due to the high risk of superimposed infection, including linezolid, meropenem, and antifungal coverage with amphotericin B. A thoracoabdominal computed tomography (CT) revealed volume overload, and a repeat TTE showed no changes compared to baseline (*Figure 1*).

During the ICU stay, the patient remained initially afebrile and without an identifiable infectious focus following the initiation of immunosuppressive therapy. However, he progressively developed multiorgan complications. Respiratory status deteriorated with the appearance of acute respiratory distress syndrome (ARDS) with bilateral alveolar infiltrates and escalating oxygen requirements, requiring invasive mechanical ventilation. Haematological pancytopenia persisted (haemoglobin 7,1 g/dL, platelets 13.000/µL, white blood cells 0,05 × 10^3^/µL), requiring repeated transfusions. Renal function also declined, with the development of acute kidney injury and oligoanuria (creatinine 1.5 mg/dL, urea 222 mg/dL) and signs of fluid redistribution, like ascites and lower limb oedema. Laboratory tests showed marked hyperphosphatemia (peak 7.1 mg/dL), with normal calcium and parathyroid hormone levels (PTH 51.6 pg/mL). Continuous renal replacement therapy (CRRT) with hemodiafiltration commenced on ICU day 6, correcting electrolytes but not fluid overload.

On ICU day 25, the patient developed a new febrile episode, prompting repeat blood cultures that identified multidrug-resistant *Pseudomonas aeruginosa* and *Enterococcus faecalis*. Targeted antimicrobial therapy was started with ceftolozane/tazobactam, cefiderocol, and ampicillin, respectively.

Despite appropriate antimicrobial therapy, the patient remained haemodynamically unstable, requiring ongoing vasopressor support (norepinephrine ∼0.25 µg/kg/min). Given the persistent clinical instability—marked by progressive respiratory deterioration and only partial reduction in inflammatory markers—a repeat contrast-enhanced thoracoabdominal CT was performed on hospital day 70 to reassess for potential infectious foci.

**Figure 2:**
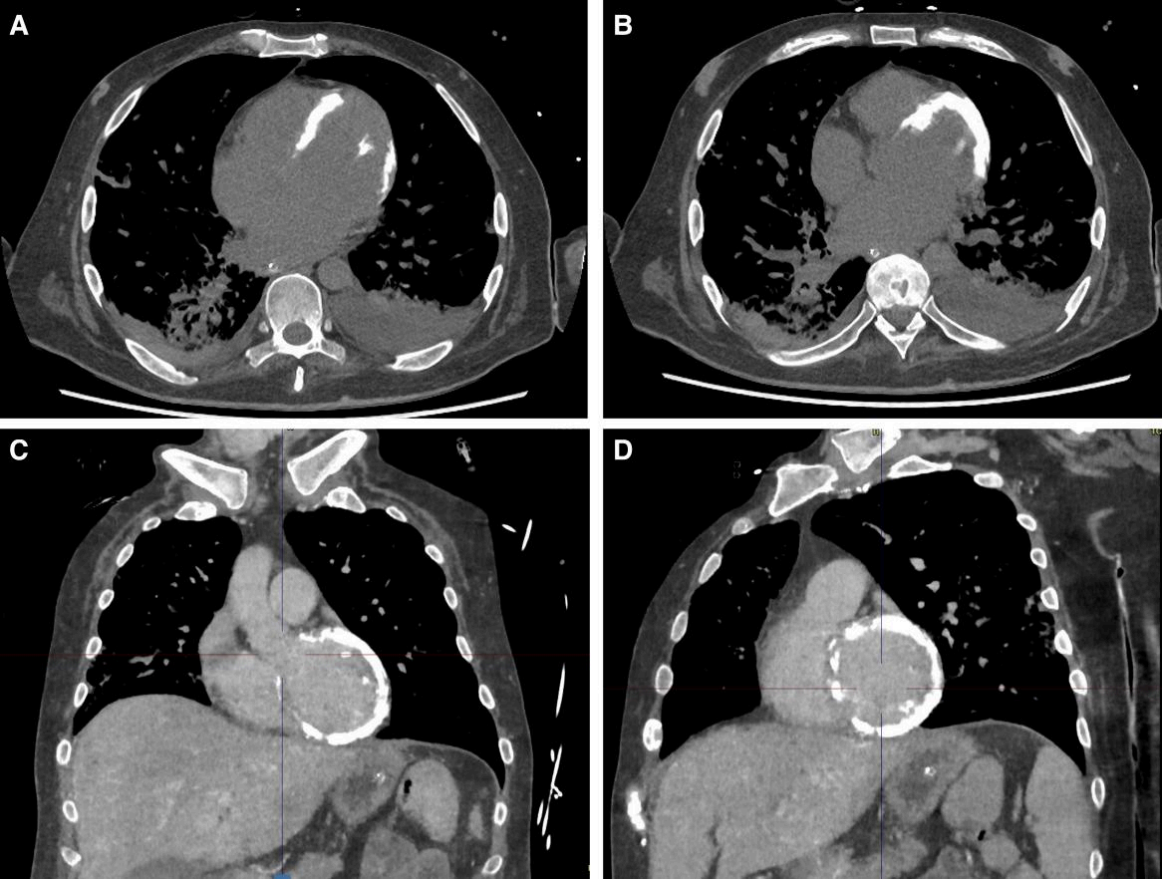
Non-contrast chest CT on day 70 of hospitalization showing extensive myocardial calcification. (*A–B*) Axial views and (*C–D*) coronal and sagittal reconstructions demonstrate diffuse epicardial and intramyocardial calcification involving the left ventricular wall. No calcification is observed in extracardiac structures. The maximum density measured was 359 Hounsfield units, consistent with calcium deposition.

Unexpectedly, the scan revealed extensive epicardial and intramyocardial calcification of the left ventricle (LV), with no evidence of calcifications in other organs. Differential diagnoses included contrast retention secondary to microvascular dysfunction in the context of suspected myocarditis. However, a non-contrast CT confirmed persistent hyperdense myocardial signal (359 Hounsfield units), consistent with calcium deposition (*Figure 2*). High-sensitivity troponin I (hs-TnI) levels were not significantly elevated (52.9 ng/mL), and the electrocardiogram (ECG) remained unremarkable.

**Figure 3:**
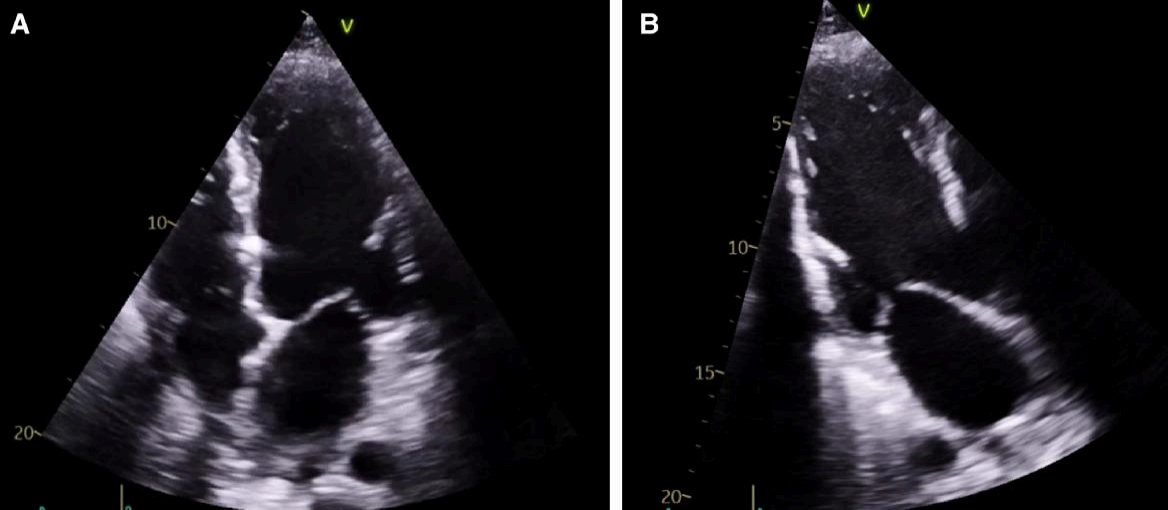
Transthoracic echocardiogram on day 77 of hospitalization. (*A*) Apical four-chamber view and (*B*) apical three-chamber view show marked hyperechogenicity in the septal and inferolateral walls, consistent with myocardial calcification. The findings correlate with impaired contractility and reduced left ventricular ejection fraction (LVEF 33%).

**Figure 4:**
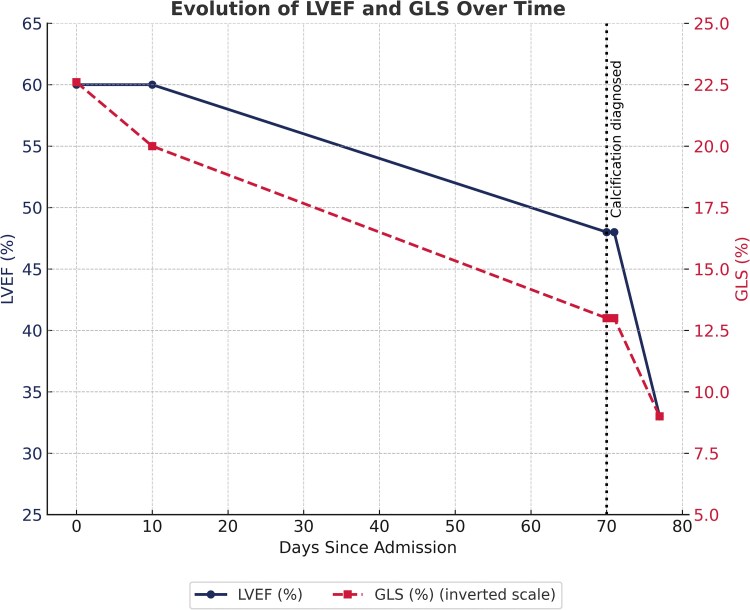
Longitudinal evolution of left ventricular function. Progressive decline in left ventricular ejection fraction (LVEF, solid line) and global longitudinal strain (GLS, dashed line; inverted scale) over time. A marked deterioration is observed after day 70, coinciding with the diagnosis of extensive myocardial calcification on CT scan.

**Figure 5:**
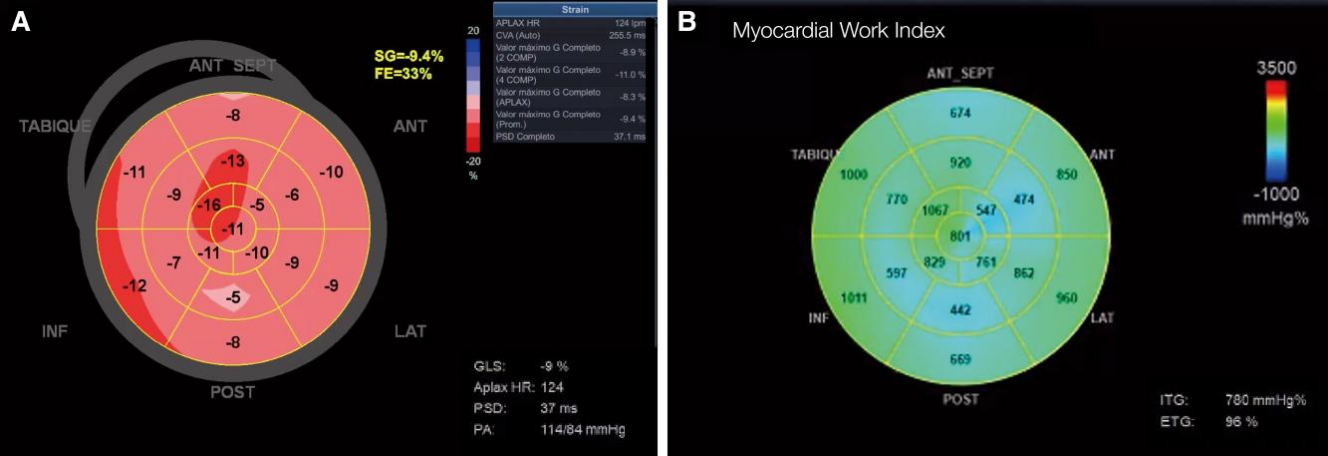
Global longitudinal strain and myocardial work analysis on day 77. (*A*) Bull's-eye plot showing severely reduced global longitudinal strain (GLS −9%), with global impairment. (*B*) Myocardial work index (GWI) analysis reveals decreased global myocardial work (780 mmHg%), especially in medial and apical regions, with preserved global work efficiency (GWE 96%).

A subsequent TTE revealed mild global systolic dysfunction (LVEF 48% by biplane Simpson), GLS −13%, and a restrictive diastolic filling pattern (E/A 3.7, E/e’ 12.5). Marked epicardial and intramyocardial hyperechogenicity was noted, predominantly in the septal and inferolateral walls, suggestive of calcific infiltration (see Supplementary material online, *Video S2*). One week later, further deterioration was observed: LVEF 33%, MAPSE 11 mm, and GLS −9% (*Figures 3, 4,* and *5A*; Supplementary material online, *Video S3*; *Table 1*). To assess intrinsic myocardial contractility independently of afterload—given that the patient remained on substantial vasopressor support (norepinephrine 0.2 µg/kg/min)—a non-invasive MW analysis was performed, integrating the measured brachial systolic arterial pressure obtained simultaneously using an automated sphygmomanometer (approximately 114 mmHg). This assessment demonstrated a markedly reduced global work index (GWI 780 mmHg%; normal range 1270–2428 mmHg%), with predominant impairment in mid and apical segments, while global work efficiency (GWE 96%) remained within the normal range, indicating reduced contractility without dyssynchrony or wasted work (*Figure 5B*).

**Table 1:** Serial echocardiographic parameters during hospital course

Echocardiographic parameter	Day 0 (admission)	Day 11 (ICU admission)	Day 70 (myocardial calcification diagnosis)	Day 77
LVEF (%)	60,5	60	48	33
MAPSE (mm)	15	15	12	9,6
E/A	0,93	1,5	3,7	0,41
E/e’	8	9	12,5	2,15
TAPSE (mm)	18	23	21	16
GLS (%)	−22,6	−22	−13	−11,5

LVEF, left ventricular function; MAPSE, mitral annular plane systolic excursion; TAPSE, tricuspid annular plane systolic excursion; GLS, global longitudinal strain.

During intensive care monitoring, the patient exhibited frequent ventricular ectopic beats, though no sustained arrhythmias were documented on continuous telemetry.

The clinical course was unfavourable, with persistent congestion despite negative fluid balances achieved with CRRT, and no possibility of respiratory recovery. The patient unfortunately died from massive alveolar haemorrhage, and therapeutic efforts were withdrawn due to futility.

## Discussion

Myocardial calcification is a relatively uncommon and poorly understood condition that can be classified into two main types: metastatic, occurring in the setting of systemic mineral-metabolism disturbances such as end-stage renal disease, hyperparathyroidism, or certain haematologic malignancies; and dystrophic, developing in previously injured tissue—most classically following myocardial infarction.^1^ However, with the widespread adoption of reperfusion techniques and the consequent reduction in extensive post-infarction necrosis, severe sepsis has emerged as one of the most common causes of dystrophic myocardial calcification.^2^

Cases of acute or subacute-onset myocardial calcification have been reported in patients with severe sepsis, multiorgan failure, and prolonged catecholamine support, sometimes appearing in less than two weeks from presentation.^4–6^ In this context, our patient’s presentation is distinct in three respects: first, the initial systemic inflammation was driven not by infection but by CRS following chemotherapy, representing the first published report of extensive myocardial calcification in association with CRS; second, the rapidity and severity of calcific deposition—clearly evident on CT by day 70; and third, the use of advanced echocardiographic techniques (GLS and non-invasive MW), which enabled non-invasive characterization of progressive ventricular dysfunction from its earliest stages.

CRS is a severe immunologic complication defined by dysregulated immune activation and massive cytokine release—especially IL-6—and clinically manifests with fever, hypotension, hypoxia, and multiorgan dysfunction.^3^ Although most often described after CAR-T cell therapy, similar reactions have been reported with monoclonal antibodies, including gemtuzumab ozogamicin, an anti-CD33 agent widely used in acute myeloid leukaemia.^7^ In our patient, inflammatory symptoms began within hours of drug administration—without any infectious focus or positive cultures—and IL-6 levels exceeded 1000 pg/mL, supporting the diagnosis of CRS.

Distinguishing CRS from sepsis is challenging in immunocompromised patients, as both present with fever, hypotension and organ dysfunction and may fulfil sepsis criteria by SOFA score. However, CRS typically manifests more acutely after immunomodulatory therapy, with pronounced cytokine release and no initial microbiological evidence of infection.^3^ In our case, systemic inflammation clearly preceded the later isolation of multidrug-resistant pathogens, reinforcing a primary CRS event followed by nosocomial sepsis.

**Figure 6:**
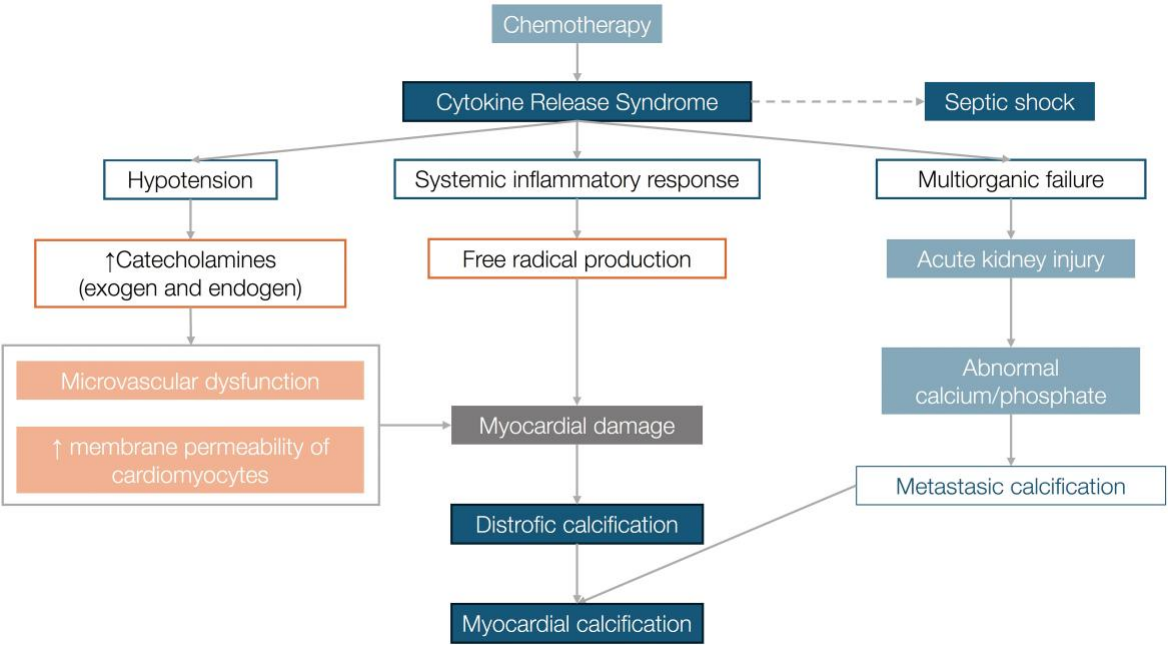
Pathophysiological mechanisms of myocardial calcification in CRS and sepsis. Chemotherapy-induced cytokine release syndrome triggers systemic inflammation, catecholamine excess, and microvascular dysfunction, leading to myocardial damage and dystrophic calcification. These processes are further aggravated by subsequent sepsis and renal failure, amplifying the inflammatory and metabolic injury to the myocardium.

From a pathophysiological perspective, myocardial calcification in this setting likely reflects a multifactorial dystrophic process (*Figure 6*). CRS induced a massive cytokine storm—with IL-6 release, endothelial injury, microvascular dysfunction and myocyte apoptosis—which was further exacerbated by prolonged catecholamine exposure and subsequent sepsis.^1,2,8^ Although our patient experienced transient hyperphosphatemia secondary to acute renal failure, serum calcium and PTH remained within normal limits; this, together with the characteristic ‘*vascular leak*’ of CRS, may have facilitated local phosphate and calcium deposition in myocardial tissue.^3^ The predominantly myocardial distribution—and absence of calcification elsewhere—supports a cardiac-specific susceptibility, possibly related to the ability of cardiac fibroblasts to differentiate into an osteogenic phenotype under sustained inflammatory stress *[Summary Figure]*.^9^

Diagnostically, the extent of calcification did not initially correspond to overt ventricular dysfunction, likely because an epicardial distribution can spare subendocardial function. GLS detected subclinical systolic impairment before a notable decline in LVEF, yet both GLS and LVEF remain load-dependent and may underestimate contractile performance under vasopressor support. To address this, we employed MW analysis, which integrates non-invasively measured blood pressure into strain curves.^10^ A markedly reduced GWI, combined with preserved GWE, confirmed intrinsic contractile impairment without mechanical dyssynchrony or wasted work.

Although specific data on MW in myocardial calcification are lacking, recent studies in chemotherapy-related cardiotoxicity and restrictive cardiomyopathies have linked lower GWI and GWE to poorer prognosis and higher cardiovascular event rates.^11,12^ In our patient, MW did not directly modify clinical management but provided a more refined understanding of disease severity and prognosis. Early identification of subtle abnormalities—such as emerging myocardial hyperechogenicity, deterioration in strain parameters or worsening MW indices—may prompt intensified monitoring and, in selected cases, earlier consideration of advanced or more aggressive therapeutic strategies, which could potentially influence outcomes when implemented at a less advanced stage of critical illness.

Cardiac magnetic resonance imaging and endomyocardial biopsy were considered for differential diagnosis—particularly to exclude infiltrative disorders such as amyloidosis or sarcoidosis—but were precluded by the patient’s haemodynamic instability, mechanical ventilation and thrombocytopenia with high bleeding risk. Consequently, the precise aetiology and mechanistic substrate of the extensive calcification remain inferential, although the temporal evolution and imaging features strongly support a predominantly dystrophic, inflammation-driven process.

No specific therapy for myocardial calcification is established. Case reports describe partial or complete recovery of ventricular function despite persistent calcific deposits, and in some instances, progressive radiologic resolution.^2^ β-Blockers have been proposed in early phases for their anti-remodelling and anti-arrhythmic effects, though clinical application must be individualized.^4^ In our patient, severe clinical deterioration limited further therapeutic options.

In summary, this case highlights CRS as a potential trigger for rapid, extensive myocardial calcification and underscores the value of advanced echocardiographic techniques (GLS and MW) for early detection of ventricular dysfunction in critically ill patients where conventional imaging and invasive diagnostics may be unfeasible. Given the multiorgan involvement and the intrinsic severity of these patients, a structured, close follow-up of ventricular function is strongly recommended.

## Conclusion

Systemic inflammatory response can significantly impact the cardiovascular system, and although myocardial calcification is a rare complication, its presence is associated with high morbidity and mortality. In this case, the rapid onset of calcification appeared to be driven by cytokine release syndrome, further exacerbated by sepsis, representing the first reported case in the literature with this dual aetiology. Additionally, this case highlights a clear correlation between the progression of calcification and functional deterioration assessed by echocardiography, where GLS and MW enabled early detection of ventricular dysfunction. These findings emphasize the need for further research into the role of inflammation in the pathophysiology of myocardial calcification and its prognostic implications in critically ill patients.

## Supplementary Material

ytag071_Supplementary_Data

## Data Availability

All data supporting the findings of this case are contained within the article and its Supplementary Information files. Additional de-identified patient data can be obtained from the corresponding author upon reasonable request.

## Lead author biography

Lucía Canales-Muñoz is a cardiology resident at La Paz University Hospital, with a strong interest in advanced cardiac imaging, arrhythmia management, and critical care cardiology. She is committed to enhancing diagnostic precision and therapeutic approaches in these specialized areas.
